# Long noncoding RNA SNHG4 promotes glioma progression via regulating miR-367-3p/MYO1B axis in zebrafish xenografts

**DOI:** 10.1007/s13577-025-01183-1

**Published:** 2025-02-14

**Authors:** Yueqing Zhang, Yongjin Wang, Yang Yang, Chunming Sun

**Affiliations:** 1 Department of Neurosurgery, The First Affiliated Hospital of Soochow University, Suzhou, 215006 People’s Republic of China; 2 Department of Neurosurgery, Huai’an Hospital of Huai’an City, Huai’an, 223200 People’s Republic of China

**Keywords:** Glioma, LncRNA SNHG4, MiR-367-3p, MYO1B, Zebrafish xenograft

## Abstract

Glioma is one of the most malignancy and prevalent tumor in the human central nervous system, which is associated with severe morbidity and high mortality. Numerous studies have explained the clear correlation between abnormal expression of lncRNA and progression of Glioma. LncRNA small nucleolar RNA host gene 4 (SNHG4) have been proved to play oncogenesis roles in various tumors, however, the underlying mechanism remains to be explored deeply. In this study, by analysis of the public database, we found that SNHG4 was upregulated in multiple cancer tissues, including glioma. Subsequently, the functional roles of SNHG4 were investigated, and we found that knockdown of SNHG4 remarkedly inhibited cell proliferation, migration. While, overexpression of SNHG4 enhanced these functions of glioma cells in vitro. Meanwhile, as the in vivo tool, zebrafish xenograft model was used to verify the functions of SNHG4 in glioma cells. Mechanically, we identified that SNHG4 or MYO1B could bind with miR-367-3p by the luciferase reporter assays. Furthermore, the rescue experiments showed that the inhibition of miR-367-3p or the expression of MYO1B partially rescue the inhibition effects of SNHG4 in glioma cells. Our study reveals that SNHG4 promotes the proliferation, migration of glioma via regulating miR-367-3p/MYO1B axis.

**Supplementary Information:**

The online version contains supplementary material available at 10.1007/s13577-025-01183-1.

## Introduction

Glioma is one of the most common malignant brain tumors with high mortality [1, 2]. About 49% of brain tumors are glioblastomas, and 30% are diffusely infiltrating lower-grade gliomas [2]. The grades of glioma were classified by the World Health Organization, including Grade I (pilocytic astrocytoma), Grade II (astrocytoma, oligodastoma, etc.), Grade III (anaplastic astrocytoma, anaplastic oligodastoma, etc.) and Grade IV (glioblastoma). With grade increases, the malignant biological properties of the glioma are enhanced, and the treatment effects are not optimistic, the median survival of patients is 14.6 months [3]. Though the progress in treatment and strategies have made, including incorporating surgery, stereotactic radiotherapy, immunotherapy and new chemotherapy drugs, the prognosis of patients has not been obviously improved [1, 4–7]. Therefore, it is essential to research the potential mechanisms to identify new diagnostic and therapeutic targets for glioma carcinogenesis.

Recently, a number of studies have identified that non-coding RNAs are involved in glioma progression. The sequencing of human genome revealed that that only about 2% of the total genomic sequence encode proteins, and over 98% of the human genomes do not encode protein [8–10]. Long non-coding RNAs (lncRNAs) are a class of RNA molecules with lengths > 200 nucleotides with no protein-coding capacity [11]. Increasing evidence have indicated that the aberrant expression of lncRNAs functioned as important regulator for protein-coding genes at transcriptional or posttranscriptional level [12, 13], and they have been showed to play critical roles in the development and progression of human tumors, such as growth, migration, invasion [12–19]. In the progression of glioma, some lncRNAs, which are significantly up-regulated, act as oncogenes. Such as lncRNA COX10-AS1 was significantly increased in glioma tissues and cell lines and promoted cell proliferation, migration and invasion by competitively binding miR-1-3p to regulate ORC6 expression [20]. LncRNA LUCAT1 promotes glioblastoma growth by enhancing HIF1α activity [21]. LncRNA PSMB8-AS1 enhanced BCAT1 expression by competitively binding to miR-382-3p to promote the migration and proliferation [22]. Numerous studies have revealed that some lncRNAs could be as the novel prognostic biomarker of glioma. Such as lncRNA RARA-AS1 [23], MALAT1 [24], lncRNA CAI2 [25]. SNHG4 is located at 5q31.2 and belongs to the family of small nucleolar RNA host genes (SNHGs), which participates in various human biological processes. It has been reported to play an oncogenic role in multiple cancers, including colorectal cancer [26], lung cancer [27], hepatocellular carcinoma [28], prostate cancer [12]. In glioblastoma, SNHG4 regulates c-Met expression by sponging miR-138 to promote the cell proliferation [29]. While, the function of SNHG4 on migration and the underlying mechanism of SNHG4 in glioma progression is still unclear.

MicroRNAs (miRNAs) are small non-coding RNAs that could bind with the 3’-untranslated region (3’-UTR) of the target messenger (mRNA) to negatively regulate gene expression at a post-transcriptional, thus degrading the target mRNA, inhibiting gene translation [30, 31]. Some miRNAs, act as oncogenes or tumor suppressors, play important roles in the development and progression of human cancers [32, 33]. MiR-99a-5p inhibits the progression of bladder cancer by targeting FZD8 [34]. MiR-502-5p plays an inhibitory role in the progression of gastric cancer through targeting MIBP [35]. MiR-1-3p inhibits the progression of Glioma by regulating ORC6 expression [20]. MiR-4524b-5p attenuates the proliferation of glioblastoma targeting ALDH1A3 via PI3K/AKT/mTOR pathway [36].

Mechanistically, lncRNAs act as competing endogenous RNAs (ceRNAs) to bind with microRNAs, thus further regulating the expression of their target genes [37, 38]. CeRNAs networks have been reported to play important role in many cancers. LncRNA PRKCA-AS1, as a ceRNA, promotes lung cancer cell proliferation and migration by sponging miR-508-5p to regulate S100A16 expression [39]. LINC02298 promotes malignancy of hepatocellular carcinoma by binding with miR-28-5p to regulate CCDC6 expression [40]. LncRNA-MIAT acts as a ceRNA to promote thyroid cancer progression by binding with miR-150-5p to regulate EZH2 expression [41].

In our research, the main purpose was to explore the roles and mechanism of SNHG4 in glioma. Functional experiments showed that SNHG4 promoted the cell proliferation and metastasis of glioma in vitro and in vivo. Mechanically, our studies showed that SNHG4 could bind with miR-367-3p and inhibited its expression. And the target gene of miR-367-3p was MYO1B was proved. Rescue assays showed that knockdown of SNHG4 inhibited glioma cell proliferation, migration, while the inhibitory effects were impaired by inhibiting miR-367-3p or overexpression of MYO1B. In conclusion, our study elucidates mechanism that SNHG4 promotes progression of glioma through miR-367-3p/MYO1B axis, which may contribute to find the potential diagnostic and therapeutic target of glioma patients.

## Materials and methods

### Online cancer database analysis

The expression levels of SNHG4 between tumor and normal tissues in multiple human cancers were analyzed by UALCAN website (https://ualcan.path.uab.edu/). The correction between SNHG4 expression levels and over survival of glioma patients was analyzed by Gene Expression Profiling Interactive Analysis (GEPIA) (http://gepia.cancer-pku.cn/). The nuclear or cytoplasmic localization of SNHG4 was analyzed by lncRNA subcellular localization predictor website (lncLocator) (http://www.csbio.sjtu.edu.cn/bioinf/lncLocator/). The specific primer for reverse transcription of miRNA to cDNA and primer for qPCR to detect the expression of miRNA were designed through miRNA Primer Design Tool (http://genomics.dote.hu:8080/mirnadesigntool/). The StarBase public database (https://rnasysu.com/) was used to predict the miRs which could bind with SNHG4. The target genes of miR-367-3p were analyzed using miRDB database (https://mirdb.org/).

### Cell culture

The human glioma cells (Ln229, U87, U251) and the human normal Brain astrocyte cell line (SVG) were obtained from Institute of Biochemistry and Cell Biology of Chinese Academy of Science (Shanghai, China). All cells were cultured in DMEM with 10% fetal bovine serum and 100 U/mL penicillin and 100 mg/mL streptomycin. The cells were cultured in a humidified atmosphere with 5% CO2 at 37 ℃.

### RNA exaction and quantitative real-time PCR

Total RNA was exacted from cultured cells using TRIzol reagent (Invitrogen, CA, USA), according to the manufacturer’s protocols. Random primers were used to reverse transcribed to cDNA of the total RNA using PrimeScript RT kit (Takara, Dalian, China). The conditions were as follows: 42 °C for 2 min, 25 °C for 5 min, 42 °C for 30 min and 85 °C for 5 min. The expression levels of different gene in the products were detected by quantitative real-time PCR (qRT-PCR) using SYBR Green Master Mix kit (Takara, Dalian, China). The conditions were as follows: 95 °C for 5 min for 1 cycle, and 95 °C for 10 s and 60 °C for 30 s for 40 cycles. Glyceraldehyde 3-phosphate dehydrogenase (GAPDH) was applied as internal reference for lncRNA and mRNA.

For reverse transcription of miRNA, the specific primers were designed to use. The stem-loop RT primer and the primers for qPCR assay were designed through miRNA Primer Design Tool (http://genomics.dote.hu:8080/mirnadesigntool/). Through the website, the information was obtained as follows: the stem-loop oligo for the reverse transcription step, the universal reverse primer, the sequence specific forward primer for qPCR assay [42]. The result of design miRNAs primers for stem-loop RT and qPCR assay through miRNA Primer Design Tool are shown in Supplementary Fig. S1. The miRNA sequences of stem-loop RT primer are listed in Table 1. Then, the miRNAs were transcribed into cDNA using miRNA specific stem specific stem loop-RT primer and Hifair^®^ II 1st Strand cDNA Synthesis Kit (YEASEN, Shanghai, China). The reaction system included two parts, remove residual genomic DNA and RT reaction, which is shown in Tables 2 and 3. The remove residual genomic DNA reaction was incubation at 42 ℃ for 2 min. The reverse transcription reaction was incubation at 25 ℃ for 5 min, 42 ℃ for 30 min, and 85 ℃ for 5 min. Then, the expression levels of miRNAs were detected by quantitative real-time PCR (qRT-PCR) using SYBR Green Master Mix kit (Takara, Dalian, China). And U6 was used as internal control for detection of miRs. All data are analyzed by 2^−∆∆Ct^ methods. The primer sequences for qPCR are listed in Table 4.

**Table 1:** The sequences of stem-loop RT primer

miRNA	Stem-loop RT primer
miR-409-3p	GTTGGCTCTGGTGCAGGGTCCGAGGTATTCGCACCAGAGCCAACAGGGGT
miR-4500	GTTGGCTCTGGTGCAGGGTCCGAGGTATTCGCACCAGAGCCAACAAGAAA
miR-495-3p	GTTGGCTCTGGTGCAGGGTCCGAGGTATTCGCACCAGAGCCAACAAGAAG
miR-32-5p	GTTGGCTCTGGTGCAGGGTCCGAGGTATTCGCACCAGAGCCAACTGCAAC
miR-363-3p	GTTGGCTCTGGTGCAGGGTCCGAGGTATTCGCACCAGAGCCAACTACAGA
miR-367-3p	GTTGGCTCTGGTGCAGGGTCCGAGGTATTCGCACCAGAGCCAACTCACCA

**Table 2:** gDNA digestion reaction system

Component	Volume
RNase-free H_2_O	To 10 μL
5 × gDNA Digester Mix	2 μL
Total RNA (1000 ng)	Volume calculated based on RNA concentration

**Table 3:** Reverse transcription reaction system

Component	Volume
RNase-free H_2_O	To 20 μL
Previous reaction solution	10 μL
5 × Hifair^®^ II Buffer plus	2 μL
Hifair^®^ II Enzyme Mix	2 μL
Gene Specific Primers (2 μM)	1μL

**Table 4:** Primer sequences for qPCR

Gene	Forward primer (5′−3′)	Reverse primer (5′−3′)
SNHG4	GCAGATGCAACTGCAGGATGAC	TGTCATTCAGGTGAAGGCATCTG
GAPDH	CAGCCTCAAGATCATCAGCA	GTCTTCTGGGTGGCAGTGAT
miR-409-3p	GTGGAATGTTGCTCGGTGA	GTGCAGGGTCCGAGGT
miR-4500	GTTTGGGGTGAGGTAGTAG	GTGCAGGGTCCGAGGT
miR-495-3p	GTTTGGAAACAAACATGGTGCA	GTGCAGGGTCCGAGGT
miR-32-5p	GTGGGGTATTGCACATTACTAA	GTGCAGGGTCCGAGGT
miR-363-3p	GGGAATTGCACGGTATCCA	GTGCAGGGTCCGAGGT
miR-367-3p	GTTGGGAATTGCACTTTAGCAA	GTGCAGGGTCCGAGGT
MYO1B	ATTGTGATTGCCGCCTGGTA	TGCTTCAGTTCCCGCAGAAT
U6	GCTTCGGCAGCACATATACTAAAAT	CGCTTCACGAATTTGCGTGTCAT

### Cell transfection

We used Lipofectamine 2000 (Invitrogen, USA) to perform the cell transfection assays. The small interfering RNAs (siRNAs) against SNHG4 (si1-SNHG4, si2-SNHG4, si3-SNHG4), negative control (NC), miR mimics and inhibitor (NC mimics, miR-367-3p mimics, NC inhibitor, miR-367-3p inhibitor), over-expression plasmids (pcDNA3.1, pcDNA3.1-SNHG4, pcDNA3.1- MYO1B) were synthesized from General Biosystems (China). And the sequences are listed in Table 5.

**Table 5:** The sequences of siRNAs

Gene	Forward primer (5′−3′)	Reverse primer (5′−3′)
si1-SNHG4	GUGACACCAAGAUAAGUAATT	UUACUUAUCUUGGUGUCACTT
si2-SNHG4	GGCUGUGUAAGUUGAUAAATT	UUUAUCAACUUACACAGCCTT
NC	UUCUCCGAACGUGUCACGUTT	ACGUGACACGUUCGGAGAATT
miR-367-3p mimics	AAUUGCACUUUAGCAAUGGUGA	UCACCAUUGCUAAAGUGCAAUU
NC mimics	UCACAACCUCCUAGAAAGAGUAGA	UCUACUCUUUCUAGGAGGUUGUGA
miR-367 inhibitor	UCACCAUUGCUAAAGUGCAAUU	
NC inhibitor	UCUACUCUUUCUAGGAGGUUGUGA	

### Cell Counting Kit-8 (CCK-8) assay

The Cell Counting Kit-8 (CCK-8, DOJINDO, Japan) was used to assess cell proliferation. Briefly, a density of 2000 cells that were transfected with siRNAs, plasmids or mimics, inhibitor per well, were seeded at in 96-well plates. After incubation for 6 h at 37 ℃, 10 μL CCK-8 reagent was added into each well that containing 100 μL medium. And 2 h later, the optional density was detected at 450 nm by using a microplate reader (BioTek Elx800, USA), as the 0 h values. Subsequently, the optional density was measured every 24 h from 0 to 96 h according to the manufacturer’s instructions.

### Transwell migration assay

Migration assays were performed using Transwell chamber inserts (8 μm pore size, Corning, NY, USA). The transfected cells were harvested and suspended in serum-free medium. A density of 3–4 × 10^4^ cells per well were seeded into the upper chambers of transwell plates. And the 800 μL medium containing 10% FBS was added bottom chamber of insert. Following culture for 24 h at 37 ℃, the surface cells of the upper membrane were removed, the cells of lower membrane were fixed with methanol for 10 min and stained with 0.1% crystal violet for 10 min at temperature. Then, the cells were imaged under the inverted microscope (10X) and counted using ImageJ software.

### Transcriptome sequencing

Total RNAs were isolated and extracted from Ln229 cells with si-SNHG4 and NC cells. Then the concentration of RNAs were measured with a NanoDrop 2000 (Thermo Scientific, USA). Subsequently, the transcriptome sequencing assay was performed and the data was analyzed.

### Luciferase reporter assay

The potential binding sites of SNHG4 or MYO1B mRNA 3’ UTR with miR-367-3p were predicted by StarBase (https://starbase.sysu.edu.cn/). The fragments of WT-SNHG4, MUT-SNHG4 and WT- MYO1B 3’ UTR, MUT-MYO1B 3’ UTR that containing miR-367-3p binding sites were synthesized and cloned into pGL3-basic vector. The reporter plasmids and miR-367-3p mimics or NC mimics were co-transfected into HEK-293 T cells using Lipofectamine 2000 reagents. After 48 h, the luciferase activity was measured using a luciferase assay kit (Promega, UAS) according to the manufacturer’s protocols.

### Western blot assay

The total protein isolated from the transfected cells by using Radio Immunoprecipitation Assay Lysis buffer (RIPA, Beyotime, China) containing phenylmethylsulfonyl fluoride (PMSF, Beyotime Biotechnology, Shanghai, China). The protein lysates were separated by 10% sodium dodecyl sulfate–polyacrylamide gel electrophoresis (SDS-PAGE). And then the proteins were electro-transferred on polyvinylidene fluoride membranes (PVDF, Millipore, Schwalbach, Germany). After that the members were blocked with 5% non-fat milk for 2 h at room temperature. Subsequently, the membranes were put into primary antibody (Abcam, UK), which for MYO1B (1:1000) or GAPDH (1:10,000) at 4 ℃ overnight, and GAPDH antibody was used as control. After that, the membranes were put into the secondary antibody (1:5000, BOSTER, China) for 2 h at room temperature. After washing, the protein signals were visualized via BeyoECL plus kit (Beyotime, China).

### Zebrafish xenograft models

The adult Tg(fli1a: EGFP) zebrafish were used in in this study, maintaining at 28 ℃ and 14 h–10 h light–dark cycle in a fish auto culture system (Haishen, China). The embryos of zebrafish were harvested and cultured into 10% Hank’s solution, which contained (in mM) 140 NaCl, 5.4 KCl, 0.25 Na2HPO4, 0.44 KH2PO4, 1.3 CaCl2, 1.0 MgSO4 and 4.2 NaHCO3 (pH 7.2). The method of constructing zebrafish xenografts according the reported article [43]. The cells that transfection with si-SNHG4 were collected and washed with PBS for two times. Then, the cells were labeled with CM-DiI (Invitrogen, USA) at 37 ℃ for 5 min, following by 15 min at 4 ℃. Next, the stained cells were washed with PBS for three time, and we examined the labeling of fluorescence by stereotype microscopy (MVX10, Olympus, Japan).

Before injection, the 48 h postfertilization (hpf) zebrafish larvae were fixed with 1.2% low-melting gel (Promega, USA). About 400 stained cells were injected into the perivitelline space (PVS) of zebrafish under a microinjector (Picosprizer III, USA), then the xenografts were cultured at 34 ℃. At 1 day post-injection (dpi), the xenografts with similar sizes of fluorescence areas were selected by stereotype microscopy (MVX10, Olympus, Japan) among them for further research, and cultured at 34 °C until the end of the experiment. At 4 dpi, the yolk and trunk of zebrafish xenografts were imaged under a stereotype microscope (MVX10, Olympus, Japan) or confocal microscope using a 20X water-immersion objective (Fluoview 3000, Olympus, Japan), which the resolution was 1600 × 1200 (MVX10) or 1024 × 1024 pixels. The analysis of images by ImageJ software after knockdown of SNHG4 in glioma cells. The valuation criteria of growth and metastasis was that the fluorescence area of the yolk and trunk of zebrafish larvae.

### Statistical analysis

All the statistical data were analyzed using unpaired Student’s *t*-test or Tukey’s multiple comparisons tests after One-way ANOVA, and their figures were generated by GraphPad Prism 8.0 software. ImageJ software was used to quantify the florescent area of zebrafish larvae. All the repetitive experiments were repeated three times. All results were presented as the mean ± SD. Each experiment was independently replicated three times. *: *P* < 0.05, **: *P* < 0.01, ***: *P* < 0.001 were considered statistically significant.

## Results

### SNHG4 is upregulated in glioma tissues and associated with prognosis of patients

**Fig. 1:**
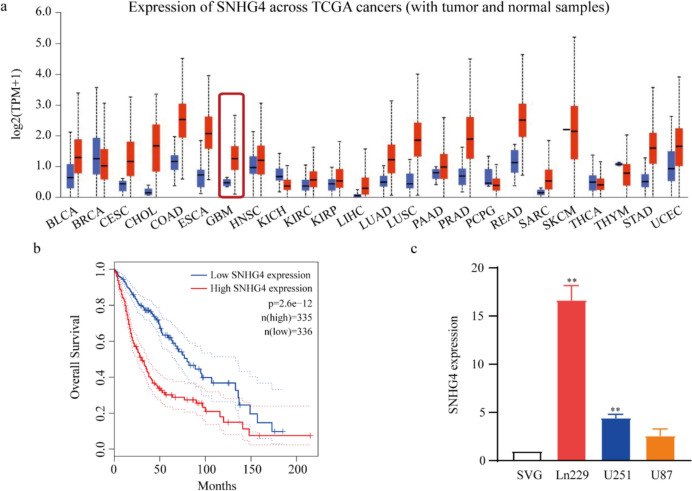
SNHG4 was upregulated in glioma tissues and cell lines and ssociated with prognosis of patients. **a** The expression level of SNHG4 in multiple cancer tissues was analyzed by UALCAN website comparing to normal tissues, including glioma tissues. **b** The correlation between overall sruvival of glioma patients and high SNHG4 expression was analyzed by Kaplan–Meier plotter. **c.** The expression level of SNHG4 was measured by qRT-PCR in human glioma cell lines (Ln229, U251,U87) compared with the normal brain astrocyte cell line (SVG) (*P* = 0.0090 and 0.0099). **: *P* < 0.01

The expression levels of SNHG4 in TCGA database were analyzed using UALCAN website, including multiple tumor tissues and normal tissues. The data showed that the SNHG4 expression was upregulated in many cancers compare with normal tissues, including glioma (Fig. 1a). Subsequently, GEPIA dataset analysis revealed that high expression of SNHG4 in glioma tissues had shorter over survival than those with low expression of SNHG4 (Fig. 1b). Furthermore, we detected the expression of SNHG4 by qRT-PCR in glioma cell lines (Ln229, U251 and U87) and the human normal brain astrocyte cell line (SVG). Among these cell lines, we found SNHG4 were highly expressed in the Ln229 and U251 cell lines, and the values were 4.5 ~ 16.7-fold higher than that of the SVG line (Fig. 1c). These results indicated that SNHG4 is upregulated in glioma and associated with poor prognosis of glioma patients.

### *SNHG4 promotes the proliferation and migration of glioma cells *in vitro

**Fig. 2:**
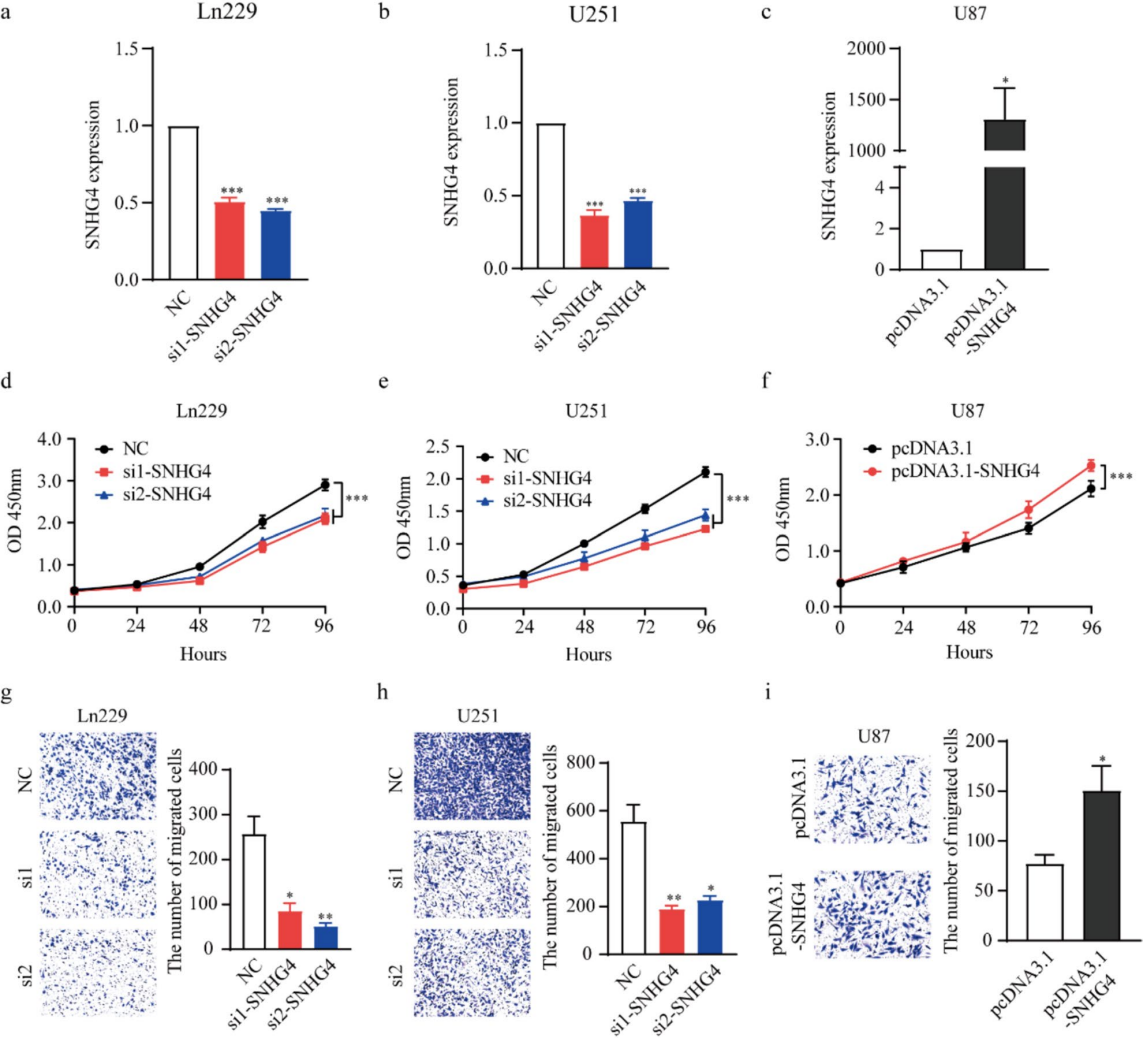
SNHG4 promoted the proliferation and migration of glioma cells in vitro.** a** The knockdown efficiency of SNHG4 was detected in Ln229 cells by qRT-PCR assay when transfection with si1-SNHG4, si2-SNHG4 and NC (*P* = 0.0027 and 0.0002).** b** The knockdown efficiency of SNHG4 was detected in U251 cells by qRT-PCR assay when transfection with si1-SNHG4, si2-SNHG4 and NC (*P* = 0.0030 and 0.0011). **c.** The overexpression efficiency of SNHG4 was detected in U87 cells by qRT-PCR assay when transfection with pcDNA3.1-SNHG4 plasmids (*P* = 0.0492). **d, e** CCK-8 assay were used to assess the proliferation of Ln229 (D) and U251 (E) cells after knocking-down SNHG4. **f** For overexpression of SNHG4 in U87 cells, the CCK-8 assay was used to assess the cell proliferation. **g, h** Transwell assays were performed to examine cell migration in Ln229 (**g**) and U251 (**h**) cells transfected with SNHG4 siRNAs (*P* = 0.0156 and 0.0064, *P* = 0.0067 and 0.0101). **i.** Transwell assays were performed to examine cell migration in U87 cells when transfection with pcDNA3.1-SNHG4 plasmids. *: *P* < 0.05, **: *P* < 0.01, ***: *P* < 0.001

**Fig. 3:**
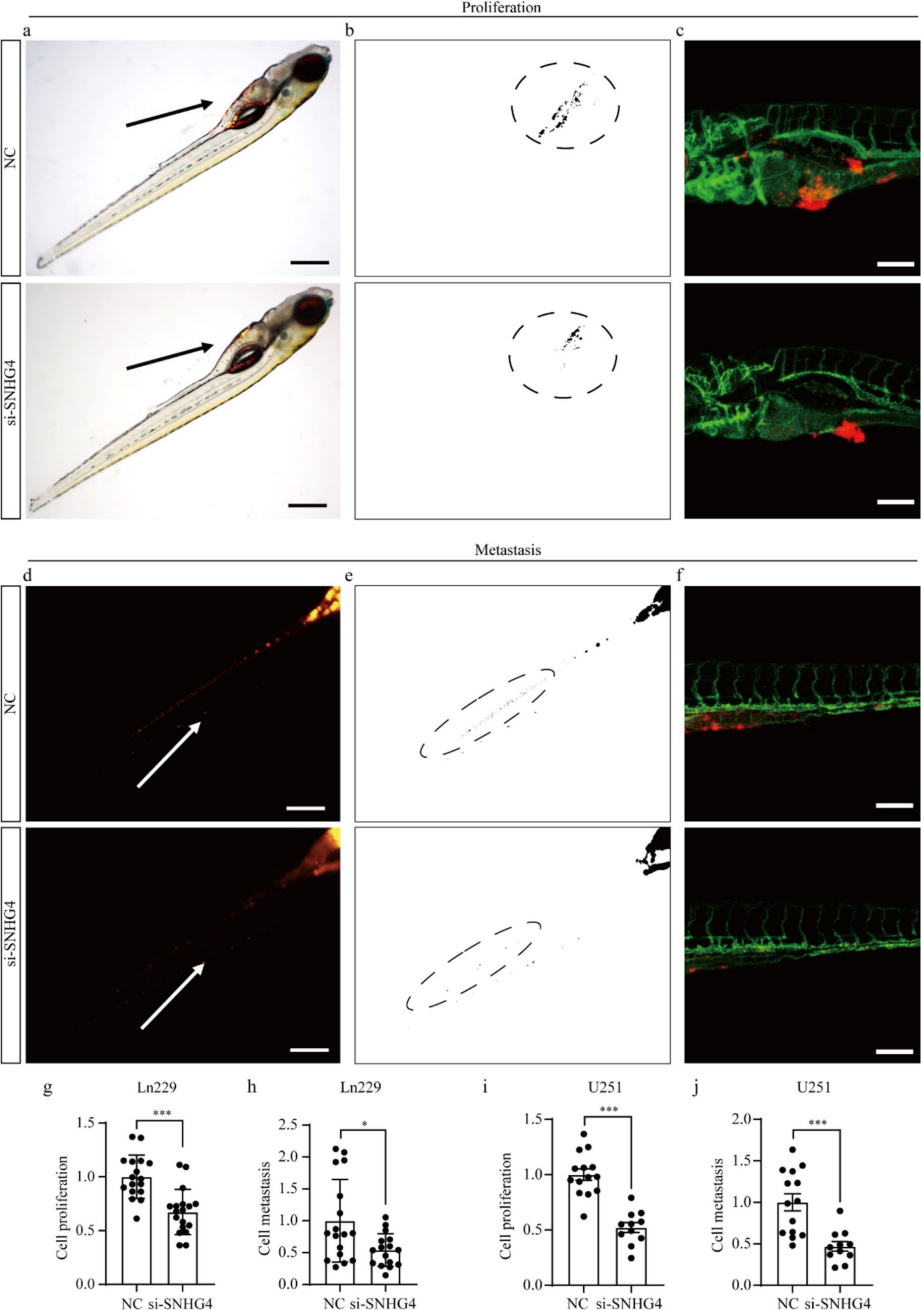
Knockdown of SNHG4 decreases the growth and metastasis of glioma cells in zebrafish xenograft. **a** The glioma cells transfected with si-SNHG4 or NC were injected into the PVS of 2-dpf (days post‑fertilization) wild type zebrafish larvae. Images of the yolk were taken by a stereomicroscope at 4 dpi. **b** The CM-DiI-positive areas in the yolk were quantified for cell proliferation. **c** The typical images were imaged by confocal microscope. **d** The glioma cells transfected with si-SNHG4 or NC were injected into the PVS of 2-dpf wild type zebrafish larvae. Images of the trunk were taken by a stereomicroscope at 4 dpi. **e** The CM-DiI-positive areas in the trunk were quantified for cell metastasis. **f** The typical images were imaged by confocal microscope. **g, h** Statistical analysis of proliferation (**g**) and metastasis (**h**) when knocking down SNHG4 in Ln229 cells (n = 17), compared with NC (*n* = 16) (*P* = 0.0001 and 0.0131). **i, j** Statistical analysis of proliferation (**i**) and metastasis (**j**) when knocking down SNHG4 in U251 cells (*n* = 14), compared with NC (*n* = 11) (*P* = 0.0002). *: *P* < 0.05, ***: *P* < 0.001. Scale: 100 μm

To verify the biological roles of SNHG4 in glioma cells, we transfected small interference RNA (siRNAs-SNHG4) to knockdown the expression of SNHG4 in Ln229 and U251 cells, and over-expression plasmids (pcDNA3.1, pcDNA3.1-SNHG4) to upregulate SNHG4 expression. The FAM and pcDNA3.1-EGFP plasmids were transfected to detect the transfection efficiency through fluorescence microscope (Fig. 2S. a-d). The efficiencies were confirmed by qRT-PCR. The knockdown efficiency of si1-SNHG4 were 49.2% and 63.3% in Ln229 and U251, respectively, compared with transfection with NC siRNA. The knockdown efficiency of si2-SNHG4 were 54.9% and 53.4% in Ln229 and U251 cells, respectively (Fig. 2a, b). The overexpression efficiency of SNHG4 is 1310 times in U87 cells, compared with transfection with pcDNA3.1 (Fig. 2c). CCK-8 assay was performed to assess cell proliferation. The results of CCK-8 assay indicated that SNHG4 knockdown inhibited the proliferation in Ln229 and U251 cells (Fig. 2d, e). While overexpression of SNHG4 has a contrasting effect in U87 cells (Fig. 2f). Transwell assay was performed and found that the migratory ability was remarkably attenuated after knockdown SNHG4 expression in Ln229 and U251 cells (Fig. 2g, h), inversely, overexpression of SNHG4 promoted migration in U87 cells (Fig. 2i). Meanwhile, in A172 cells, si-SNHG4 or pcDNA3.1-SNHG4 were transfected into A172 cells and the efficiency was detected (Fig. 3S. a, b). The function assay showed that knockdown SNHG4 expression inhibited cell proliferation and migration (Fig. 3S. c, d), inversely, overexpression of SNHG4 promoted cell proliferation and migration in A172 cells (Fig. 3S. e, f). These results indicated that SNHG4 promoted glioma progression.

**Fig. 4:**
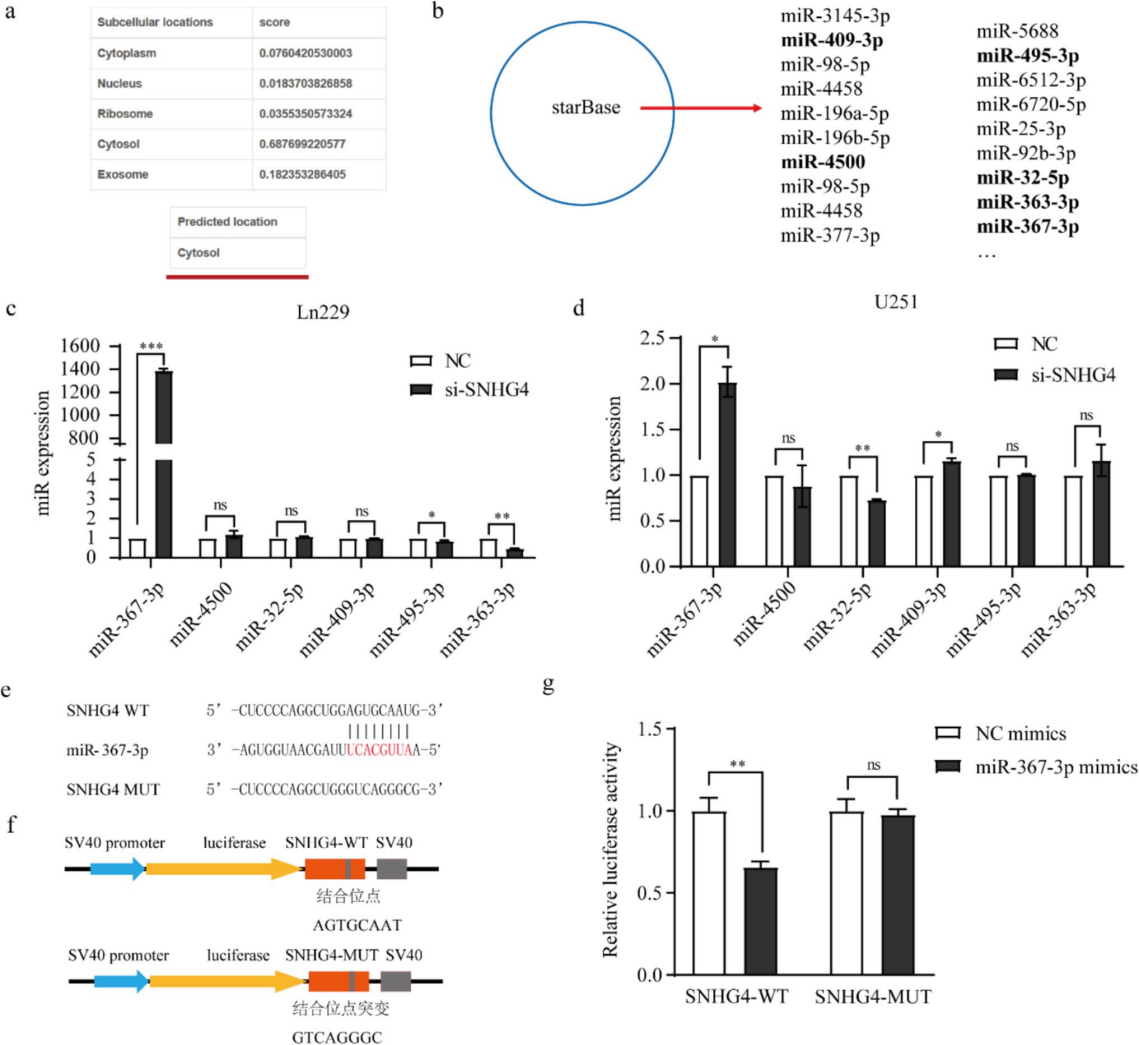
SNHG4 was a ceRNA to sponge miR-367-3p. **a** Subcellular localization of SNHG4 was analyzed by LncLocator website. **b** The potential miRNAs which might bind to SNHG4 by StarBase. **c, d** The expression levels of six miRNAs were examined when knockdown of SNHG4 in Ln229 (**c**) and U251 (**d**) cells by qRT-PCR (*P* < 0.0002 and 0.0249). **e** The potential binding site between miR-367-3p and SNHG4 was predicted by StarBase. **f** Schematic diagram of luciferase reporter plasmids construction, named pGL3-promoter-SNHG4-WT and pGL3-promoter-SNHG4-MUT. **g** Luciferase reporter assay was performed to assess the interaction between LINC01123 and miR-384 (*P* = 0.0031). *: *P* < 0.05, **: *P* < 0.01, ***: *P* < 0.01

For knockdown of SNHG4, short hairpin RNAs (shRNAs) targeting human SNHG4 were designed, synthesized and transfected into Ln229 cells. The knockdown efficiency was 97.8% (Fig. 4S. a). Then the cell proliferation and migration were detected by CCK-8 and Transwell assays. The results showed the knockdown of SNHG4 inhibited cell proliferation and migration (Fig. 4S. b, c).

**Fig. 5:**
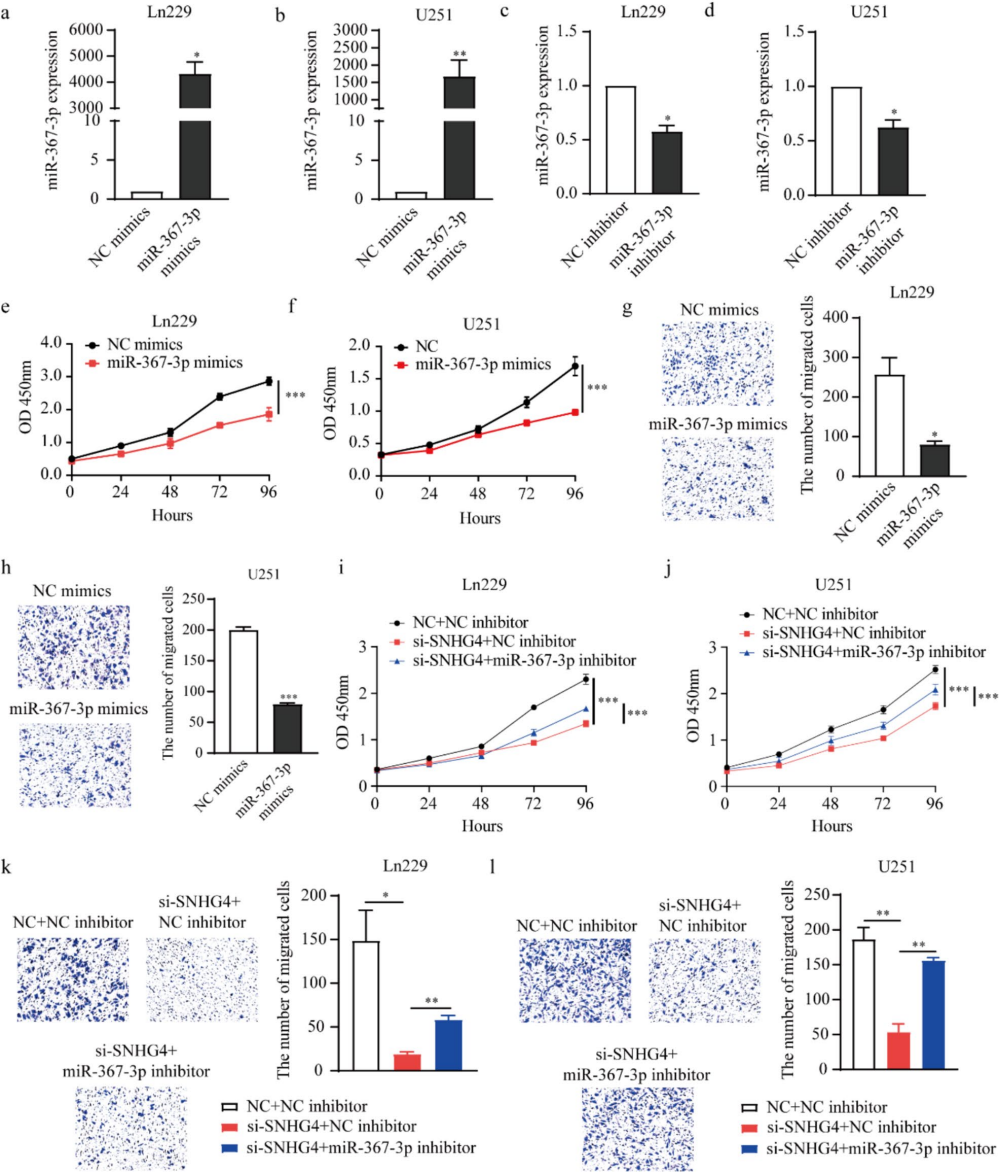
SNHG4 interacts with miR‐367-3p to regulate glioma cell proliferation and migration. **a, b** The overexpression efficiency of miR-367-3p in Ln229 (**a**) and U251 (**b**) cells when transfecting its mimics by qRT-PCR assay (*P* = 0.0106 and 0.0234). **c, d** The inhibitory efficiency of miR-367-3p in Ln229 (**c**) and U251 (**d**) cells when transfecting its inhibitor by qRT-PCR assay (*P* = 0.0163 and 0.0313). **e, f** The proliferation was assessed by CCK-8 assay when overexpression miR-367-3p in Ln229 (**e**) and U251 (**f**) cells. **g, h** The migration was assessed by Transwell assay when overexpression miR-367-3p in Ln229 (**g**) and U251 (**h**) cells (*P* = 0.0486). **i, i.** CCK-8 assay was used to evaluate cell proliferation and migration when co-transfection of miR-384 inhibitor and si-SNHG4 in Ln229 (**i**) and U251 (**j**) cells. **k, l** Transwell assays was used to evaluate cell migration when co-transfection of miR-384 inhibitor and si-SNHG4 in Ln229 (k) and U251 (l) cells (*P* = 0.0207 and 0.0022, 0.0029 and 0.0011). *: *P* < 0.05, **: *P* < 0.01, ***: *P* < 0.01

In addition, rescue experiments were performed to address the potential off-target effects. We transfected si-SNHG4 in Ln229 cells to knockdown the expression of SNHG4, meanwhile over-expressed SNHG4 expression by transfection with pcDNA3.1-SNHG4 plasmids. The results of CCK-8 assay showed that the knockdown of SNHG4 inhibited cell proliferation, while overexpression of SNHG4 partly impaired these effects in Ln229 cells (Fig. 5S. a). Transwell assay showed that the knockdown of SNHG4 inhibited cell migration, while overexpression of SNHG4 partly rescued the migration ability in Ln229 cells (Fig. 5S. b).

### *Knockdown of SNHG4 inhibits the growth and metastasis of glioma cells *in vivo

To verify the functions of SNHG4 in vivo, the zebrafish xenograft models were used in this experiment as the in vivo tools. And the transgenic zebrafish line Tg(fli1a: EGFP) which the vascular endothelial cells were labeled by EGFP was used in this experiment. The labeled cells were implanted into the PVS of 48-hpf zebrafish larvae.

At 4 dpi, the CM-DiI positive signals in yolk and trunk of zebrafish xenografts were imaged by a stereotype microscope. For cell proliferation, we imaged the area with CM-DiI positive signals of the yolk through stereotype microscopy (Fig. 3a). ImageJ software was used to quantify the area of fluorescence signals that the part circled by the dashed line, which represented the tumor cells proliferation (Fig. 3b). And the typical images were acquired by confocal microscope (Fig. 3c). For cell metastasis, we imaged the area with CM-DiI positive signals of the trunk through stereotype microscopy (Fig. 3d). ImageJ software was used to quantify the area of fluorescence signals that the part circled by the dashed line, which represented the tumor cells metastasis (Fig. 3e). And the typical images were acquired by confocal microscope (Fig. 3f). The results showed that knocking-down SNHG4 inhibited growth and metastasis of Ln229 cells, and the knockdown efficiency was 31.1% (*P* = 0.0001) and 65% (*P* = 0.0131) in growth and metastasis, respectively, compared with the NC group (Fig. 3g, h). And one dot corresponded to one zebrafish. Similarly, silencing SNHG4 inhibited the growth and metastasis of U251 cells, and the knockdown efficiency was 47.8% (*P* < 0.0001) and 53.1% (*P* = 0.0002) in growth and metastasis, respectively, compared with the NC group (Fig. 3i, j). These results demonstrate that knockdown of SNHG4 inhibits the growth and metastasis of glioma cells in vivo.

**Fig.6:**
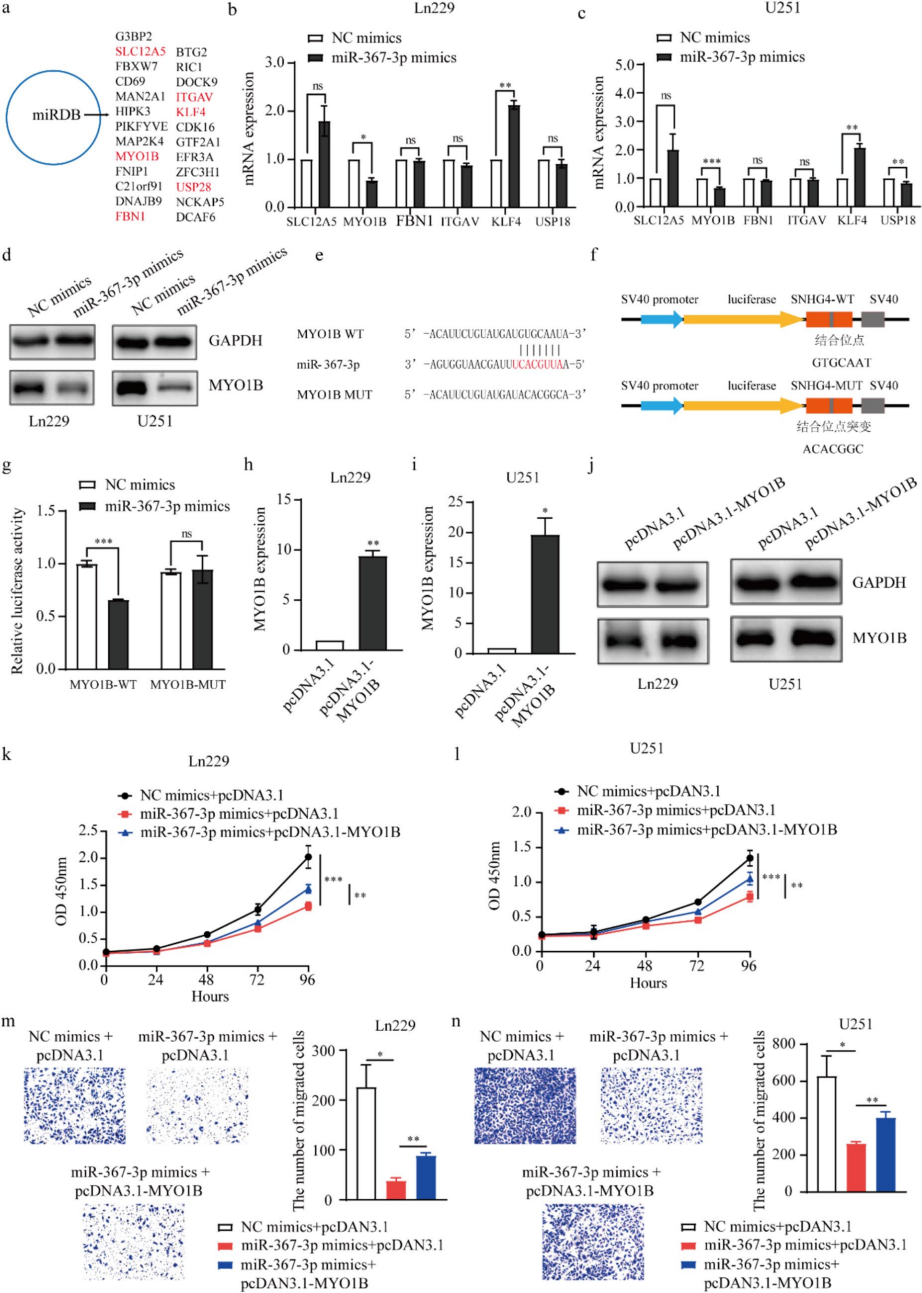
MYO1B is a target of miR-367-3p, and miR-367-3p regulates the progression of glioma through MYO1B. **a** The target genes of miR-367-3p was predicted by miRDB website. **b, c** The expression levels of target genes were detected in mRNA levels by qRT-PCR assay after overexpression of miR-367-3p in Ln229 (**b**) and U251 (**c**) cells (*P* = 0.0128 and 0.0062, 0.0002 and 0.0072). **d** The expression levels of MYO1B were detected in protein levels by western blot assay after overexpression of miR-367-3p in Ln229 and U251 cells. **e** The potential binding site between miR-367-3p and MYO1B 3’UTR was predicted by StarBase. **f** Schematic diagram of luciferase reporter plasmids construction, named pGL3-promoter-MYO1B-WT and pGL3-promoter-MYO1B-MUT. **g** Luciferase reporter assay was performed to assess the interaction between MYO1B 3’UTR and miR-367-3p (*P* = 0.0004). **h, i** The overexpression efficiency of MYO1B when transfection with pcDNA3.1- MYO1B plasmids in Ln229 (**h**) and U251 (**i**) cells at mRNA levels by qRT-PCR assays (*P* = 0.0039 and 0.0212). **j.** The overexpression efficiency of MYO1B when transfection with pcDNA3.1- MYO1B plasmids in Ln229 and U251 cells at protein level by western blot assays (*P* = 0.0039 and 0.0212). **k, l** CCK-8 assay was used to evaluate cell proliferation and migration when co-transfection of miR-367-3p mimics and pcDNA3.1- MYO1B in Ln229 (**k**) and U251 (**l**) cells. **m, n** Transwell assays were used to evaluate cell migration when co-transfection of miR-367-3p mimics and pcDNA3.1- MYO1B in Ln229 (**m**) and U251 (**n**) cells (*P* = 0.0495 and 0.0034). *: *P* < 0.05, **: *P* < 0.01, ***: *P* < 0.01

Additionally, we imaged the area with CM-DiI positive signals of the yolk through stereotype microscopy at 1, 3, 5 dpi to detect the progression of glioma cells (Fig. 6S. a). The results showed that the cell proliferation of NC group and si-SNHG4 group had no difference at 1 dpi, but at 3, 5 dpi, si-SNHG4 group was inhibited significantly, compared with NC group (Fig. 6S. b). These results demonstrated that the progression of glioma was a continuous process.

### SNHG4 inhibits miR‐367-3p expression by directly binding to miR‐367-3p

To investigate the mechanism that how SNHG4 regulates the proliferation and migration of glioma cells, we used LncLocator website (http://www.csbio.sjtu.edu.cn/bioinf/lncLocator/) to predict the subcellular localization of SNHG4. The data showed that SNHG4 distributed in both the nucleus and cytoplasm, and more abundant in the cytoplasm (Fig. 4a), which confirmed that SNHG4 could play roles in post-transcriptional level. In previous research, SNHG4 can act as a ceRNA to participate in tumor progression by sponging miRNAs [12, 28, 44–47]. To verify whether SNHG4 has this function, we used StarBase public database (https://rnasysu.com/) to predict the miRs, which target SNHG4. Among these miRs, we chose miR-409-3p, miR-4500, miR-495-3p, miR-32-5p, miR-363-3p, miR-367-3p (Fig. 4b), which repressed glioma progression [48–53]. To verify the correlation between and SNHG4, the expression of miRs were detected after silencing SNHG4 in Ln229 and U251 cells, and found only miR-367-3p showed a negative correlation, which the expression was upregulated by 1387.5 and 2 times, respectively (Fig. 4c, d). Next, we performed the luciferase reporter assay to verify that miR-367-3p bind with SNHG4. The potential binding sites were predicted StarBase public database (https://rnasysu.com/) (Fig. 4e), we constructed the SNHG4-WT and SNHG4-MUT plasmids for the luciferase reporter assay (Fig. 4f). The results indicated that the luciferase activity was inhibited of SNHG4-WT, and the inhibitory efficiency was 34.8%, while no obvious effect on that of SNHG4-MUT group (Fig. 4g). In conclusion, SNHG4 could act as the miR-367-3p sponge by directly binding to miR‐367-3p and negatively regulated its expression.

### SNHG4 interacts with miR‐367-3p to regulate glioma cell proliferation and migration

To investigate the biological function of miR-367-3p in glioma cells, we synthesized the miR-367-3p mimics to overexpression its expression. The miR-367-3p mimics and NC mimics were transected into Ln229 and U251 cells, respectively, and the overexpression efficiency was detected by qRT-PCR. The results showed that the expression of miR-367-3p was upregulated by 4334.9 (*P* = 0.0106) and 1675.7 times (*P* = 0.0234), respectively, compared with NC mimics group (Fig. 5a, b). The miR-367-3p inhibitor and NC inhibitor were transected into Ln229 and U251 cells respectively, and the inhibitor efficiency was detected by qRT-PCR. The results showed that the expression of miR-367-3p was inhibited, compared with NC inhibitor group, the inhibitory efficiency was 42.3% (*P* = 0.0163) and 37.5% (*P* = 0.0313) (Fig. 5c, d). Then, the functions on the cell proliferation and migration were assessed by CCK-8 and Transwell assays. The results of CCK-8 assay showed that cell proliferation was inhibited after overexpression of miR-367-3p in Ln229 and U251 cells (Fig. 5e, f). The results of Transwell assay showed that cell migration was inhibited after overexpression of miR-367-3p in Ln229 and U251 cells. The inhibitory efficiency was 31.4% (*P* = 0.0486) and 39.8% (*P* < 0.0001) (Fig. 5g, h). These data demonstrated that miR-367-3p could act as a suppressor in glioma cells.

To determine whether SNHG4 promoted the progression of glioma through miR-367-3p, we performed rescue experiment. The CCK-8 and Transwell assays were performed to investigate the SNHG4 and miR-367-3p interaction on the cell proliferation and migration. The results showed that the knockdown of SNHG4 inhibited cell proliferation, while inhibition of miR-367-3p partly impaired these effects in Ln229 and U251 cells (Fig. 5i, i). Cell migration was inhibited after the knockdown of SNHG4, and the inhibition efficiency was 87.2%. While inhibition of miR-367-3p partly impaired these effects in Ln229 and U251 cells, and the rescued efficiency was 67.4% (Fig. 5k, l). These results indicated SNHG4 participated in the progression of glioma through miR-367-3p.

**Fig.7:**
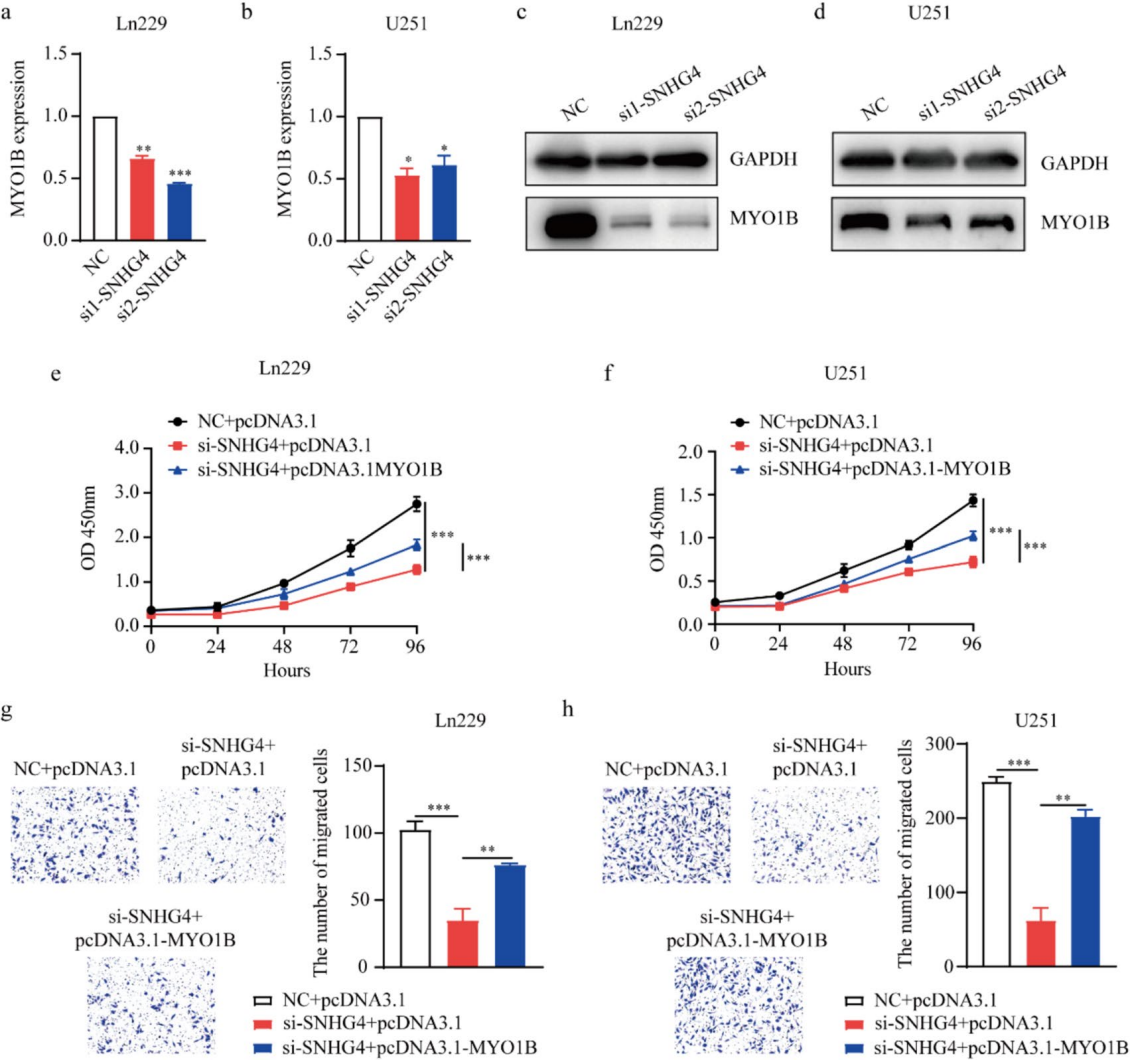
SNHG4 regulated the proliferation and migration of glioma cells via MYO1B. **a, b** The expression levels of MYO1B were examined after silencing SNHG4 in Ln229 (**a**) and U251 (**b**) cells at mRNA level by qRT-PCR assay (*P* = 0.0042 and 0.0001, 0.0126 and 0.0362). **c, d** The expression levels of MYO1B were examined after silencing SNHG4 in Ln229 (**c**) and U251 (**d**) cells at protein level by western blot assays. **e, f** CCK-8 assay was used to evaluate cell proliferation when co-transfection of si-SNHG4 and pcDNA3.1-MYO1B plasmid in Ln229 (**e**) and U251 (**f**) cells. **g, h** Transwell assay was used to evaluate cell migration when co-transfection of si-SNHG4 and pcDNA3.1-MYO1B plasmid in Ln229 (**g**) and U251 (**h**) cells (P = 0.0004 and 0.0012, 0.0004 and 0.0017). *: *P* < 0.05, **: *P* < 0.01, ***: *P* < 0.001

In addition, we detected the SNHG4 expression after transfection with miR-367-3p inhibitor, and the results showed that SNHG4 expression was up-regulated after inhibition of miR-367-3p expression (Fig. 7S. a). Then, rescue experiments were performed to address the potential off-target effects. We transfected miR-367-3p mimics in Ln229 cells to overexpress the expression of miR-367-3p, meanwhile up-regulated SNHG4 expression by transfection with pcDNA3,1-SNHG4 plasmids. The results of CCK-8 assay showed that the overexpression of miR-367-3p inhibited cell proliferation, while overexpression of SNHG4 partly repaired these effects in Ln229 cells (Fig. 7S. b). Transwell assay showed that the overexpression of miR-367-3p inhibited cell migration, while transfection with pcDNA3.1-SNHG4 partly rescued the migration ability in Ln229 cells (Fig. 7S. c). Then, we transfected miR-367-3p inhibitor in Ln229 cells to inhibit the expression of miR-367-3p, meanwhile down-regulated SNHG4 expression by transfection with si-SNHG4. The results of CCK-8 assay showed that the inhibition of miR-367-3p promoted cell proliferation, while down-regulation of SNHG4 partly impaired these effects in Ln229 cells (Fig. 7S. d). Transwell assay showed that the overexpression of miR-367-3p inhibited cell migration, while transfection with si-SNHG4 partly rescued the migration ability in Ln229 cells (Fig. 7S. e).

### MYO1B/miR-367-3p axis regulates the progression of glioma

By bioinformation analysis, the target gene of miR-367-3p was researched. We used the StarBase (https://rnasysu.com/) and miRDB (https://mirdb.org/) public database to predict the target genes of miR-367-3p. Among of them, SLC12A5, MYO1B, FBN1, ITGAV, KLF4 and USP28 were more reported to effect cell proliferation and migration in human cancers [54–59] (Fig. 6a). Based on these, we chose these genes to detect after overexpression of miR-367-3p in Ln229 and U251 cells by qRT-PCR assay. The results showed that MYO1B was more significantly down-regulated and we chose MYO1B for further research (Fig. 6b, c). At protein levels, the MYO1B expression was significantly down-regulated after transfection with miR-367-3p mimics in Ln229 and U251 cells (Fig. 6d). Additionally, the MYO1B expression was detected after inhibition of miR-367-3p, and the results showed MYO1B expression was up-regulated after transfection with miR-367-3p inhibitor (Fig. 8S). Subsequently, the potential binding sites were predicted StarBase public database (https://rnasysu.com/) between miR-367-3p and MYO1B (Fig. 6e). And we constructed the WT and MUT MYO1B 3′-UTR reporter plasmids for the luciferase reporter assay (Fig. 6f). The reporter plasmids and miR-367-3p mimics or NC mimics were co-transfected into HEK-293 T cells using Lipofectamine 2000 reagents. The results indicated that the luciferase activity was inhibited of MYO1B 3′-UTR WT, while no obvious effect on that of MYO1B 3′-UTR MUT group (Fig. 6g). These results proved that miR-367-3p negatively regulate MYO1B expression and could bind to MYO1B in glioma cells.

Then, we investigated whether miR-367-3p could involve in glioma cell proliferation and migration through MYO1B. The MYO1B expression was upregulated in Ln229 and U251 cells by transfection with pcDNA3.1-MYO1B plasmids, and the efficiency was detected in mRNA levels by qRT-PCR analysis (Fig. 6h, i). In protein levels, the expression levels of MYO1B were also upregulated after transfection with pcDNA3.1-MYO1B plasmids in Ln229 and U251 cells (Fig. 6j). The rescue experiments of cell proliferation proved that the inhibitory effects arising by the miR-367-3p mimics in glioma cells could be alleviated by the overexpression of MYO1B in Ln229 and U251 cells (Fig. 6k, l). Similarly, the rescue experiments on cell migration obtained the same impaired effects in Ln229 and U251 cells (Fig. 6m, n). In conclusion, the miR-367-3p/MYO1B axis regulates the progression of glioma cells.

### SNHG4 participates the proliferation and migration of glioma cells by regulating MYO1B expression

To investigate that SNHG4 plays roles in the progression of glioma through MYO1B-regulated pathway, we first detected the expression of MYO1B after knocking down SNHG4 in Ln229 and U251 cells in mRNA and protein levels by qRT-PCR and western blot assays. The results showed that MYO1B expression was reduced after knocking down SNHG4 in mRNA levels (Fig. 7a, b) and protein levels (Fig. 7c, d). All the western blot uncropped images were showed in the file named western blot uncropped images. Additionally, we performed the RNA sequencing to analysis KEGG pathway. And we found the MYO1B expression was down-regulated (Fig. 9S. a-c). Then, the rescue experiments were performed by co-transfecting si-SNHG4 and pcDNA3.1-MYO1B plasmid in glioma cells. The CCK-8 assay showed the suppression effects on the proliferation which were caused by silencing SNHG4 were partially counteracted by overexpression of MYO1B in Ln229 and U251 cells (Fig. 7e, f). Transwell assay showed the suppression effects on the migration which were caused by silencing SNHG4 were partially counteracted by overexpression of MYO1B in Ln229 and U251 cells (Fig. 7g, h). These results indicate that SNHG4 regulates the progression of glioma cells through the MYO1B pathway.

## Discussion

Glioma is the most common primary malignant brain tumor and is characterized by high rates of recurrence and mortality. Due to lack of effective strategies for early diagnosis for glioma patients, five-survival rate is not optimistic. Hence, it is urgent to explore the precise mechanism of the incidence and development. Numerous evidence has revealed that lncRNAs are crucial roles in the progression of human tumors and identified as diagnostic and prognostic biomarkers [60, 61]. For example, lncRNA PCAT6 acts as a promising predictor of poor prognosis for colorectal cancer [62]. LINC00467 promotes bladder cancer cell proliferation and migration via regulation of the NF-κb pathway [63]. In this study, the results showed that SNHG4 was highly expressed in glioma tissues and cells lines. Functional assays demonstrated that SNHG4 played oncogenic roles by promoting cell proliferation in vitro and in vivo. And we have proved that SNHG4 promoted cell migration of glioma in vitro and in vivo for the first time. All these data indicated that SNHG4 promoted the progression of glioma.

Numerous evidence has demonstrated that lncRNAs could act as ceRNAs through sponging miRNAs to regulate the expression of its target genes, and participate in the progression of human tumors. For example, lncRNA RAD51-AS1 promotes the progression of ovarian cancer by sponging miR-140-3p to regulate EIF5A2 expression [64]. LncRNA PVT1 serves as a ceRNA for miR-27b-3p to promote prostate cancer progression [65]. LncRNA PSMB8-AS1 acts as a sponge to bind to miR-382-3p, promoting cell proliferation and migration of glioma [22]. LncRNA DANCR promotes breast cancer cell proliferation, migration and invasion through the miR‑34c/E2F1 axis [66]. In our study, according to the subcellular RNA fraction analysis, SNHG4 was mainly located in the cytoplasm, which indicating that SNHG4 play oncogenic roles through ceRNA mechanism. Then, we proved that SNHG4 could serve as a sponge to bind to miR-367-3p by bioinformation analysis and luciferase reporter assay, which has been reported yet. And MYO1B was a target gene of miR-367-3p, which was verified by bioinformation analysis and luciferase reporter assay, which has been reported yet. Functionally, rescue experiments were performed and the results showed that the inhibitory effect of SNHG4 knockdown was impaired by inhibition of miR-367-3p on cell proliferation and migration of glioma. Overexpression of miR-367-3p or knocking down of SNHG4 inhibited cell proliferation and migration, which was rescued partly by overexpression of MYO1B. These data revealed a novel pathway that SNHG4 promoted glioma progression through miR-367-3p/MYO1B axis. All these results were shown in table S1.

In our study, zebrafish xenograft models were used as an in vivo model to verify the roles of SNHG4 on cell growth and metastasis of glioma. In previous study, the functional phenotype of lncRNAs was verified in vivo by using mouse xenograft models, however, the moused models of metastasis were rare. Based on these issues, zebrafish xenograft model was applied [67–70]. Compared with mouse models, zebrafish xenograft models have its unique advantages. First, zebrafish xenografts experiment is conducted quickly for tumor biology studies for evaluating the growth and metastasis with 4 days after transplantation, while mouse model required 2–4 weeks. Second, before injection, for zebrafish xenograft model, the cells are transfected with siRNAs and labeled by CM-DiI fluorescent dye, while for the mouse model, the cells must be transfected with short hairpin RNA (shRNA). Third, we can evaluate the growth and metastasis simultaneously, and the behavior of tumor cells are monitored with high-resolution, timely and 3-dimensional (3D) imaging in vivo based on imaging technology. Fourth, the sample demand for the experiment is small and the operating procedures is relatively simple in zebrafish xenografts. In addition, at early stages, the development of tumor cells was observed in vivo, which is impossible for mouse models. In our study, zebrafish xenograft model was used as the in vivo model to verify the biological roles and the results showed that knocking down of SNHG4 inhibited the growth and metastasis of glioma cells in vivo. The results of zebrafish xenografts were consistent with the results in mouse models based on the previous studies. These data indicate that zebrafish xenograft models could be used for tumor models for reaching the biology of cancer-related genes, which might be a reliable and effective model for human cancer research.

## Conclusions

In summary, this study verified the biological roles of SNHG4 in glioma cells in vitro and in zebrafish xenograft model, especially the function of migration, which is the first time confirmed. Mechanically, SNHG4 participated in the progression of glioma through the miR-367-3p/MYO1B axis, which was a novel signaling pathway of SNHG4. The SNHG4/miR-367-3p/MYO1B axis might provide a novel approach for the treatment of glioma patients, which might be used as the potential prognostic and therapeutic biomarkers.

## Supplementary Information

Below is the link to the electronic supplementary material.

Supplementary file1 (DOCX 4608 KB)

## Data Availability

The data that support the findings of this study are available from the corresponding author upon reasonable request.

## Abbreviations

LncRNA: Long non-coding RNA
SNHG4: Small nucleolar RNA host gene 4
MiRNA: MicroRNA
3’-UTR: 3’-Untranslated region
mRNA: Messenger
CeRNA: Competing endogenous RNA
GEPIA: Gene Expression Profiling Interactive Analysis
qRT-PCR: Quantitative real-time PCR
GAPDH: Glyceraldehyde 3-phosphate dehydrogenase
siRNA: Small interfering RNA
NC: Negative control
CCK-8: Cell Counting Kit-8
RIPA: Radio Immunoprecipitation Assay Lysis buffer
PMSF: Phenylmethylsulfonyl fluoride
SDS-PAGE: Sodium dodecyl sulfate–polyacrylamide gel electrophoresis
PVDF: Polyvinylidene fluoride membranes
PVS: Perivitelline space
Hpf: Hour postfertilization
Dpi: Day post injection
Dpf: Day post fertilization
