# Supplementary material for: Long noncoding RNA SNHG4 promotes glioma progression via regulating miR-367-3p/MYO1B axis in zebrafish xenografts
Source: Hum Cell. 2025 Feb 14;38(2):53. doi: 10.1007/s13577-025-01183-1 (PMC11828807; doi:10.1007/s13577-025-01183-1)
Supplement: Supplementary file 1 — Supplementary file1 (DOCX 4608 KB) [file 13577_2025_1183_MOESM1_ESM.docx]

**Figure legends**


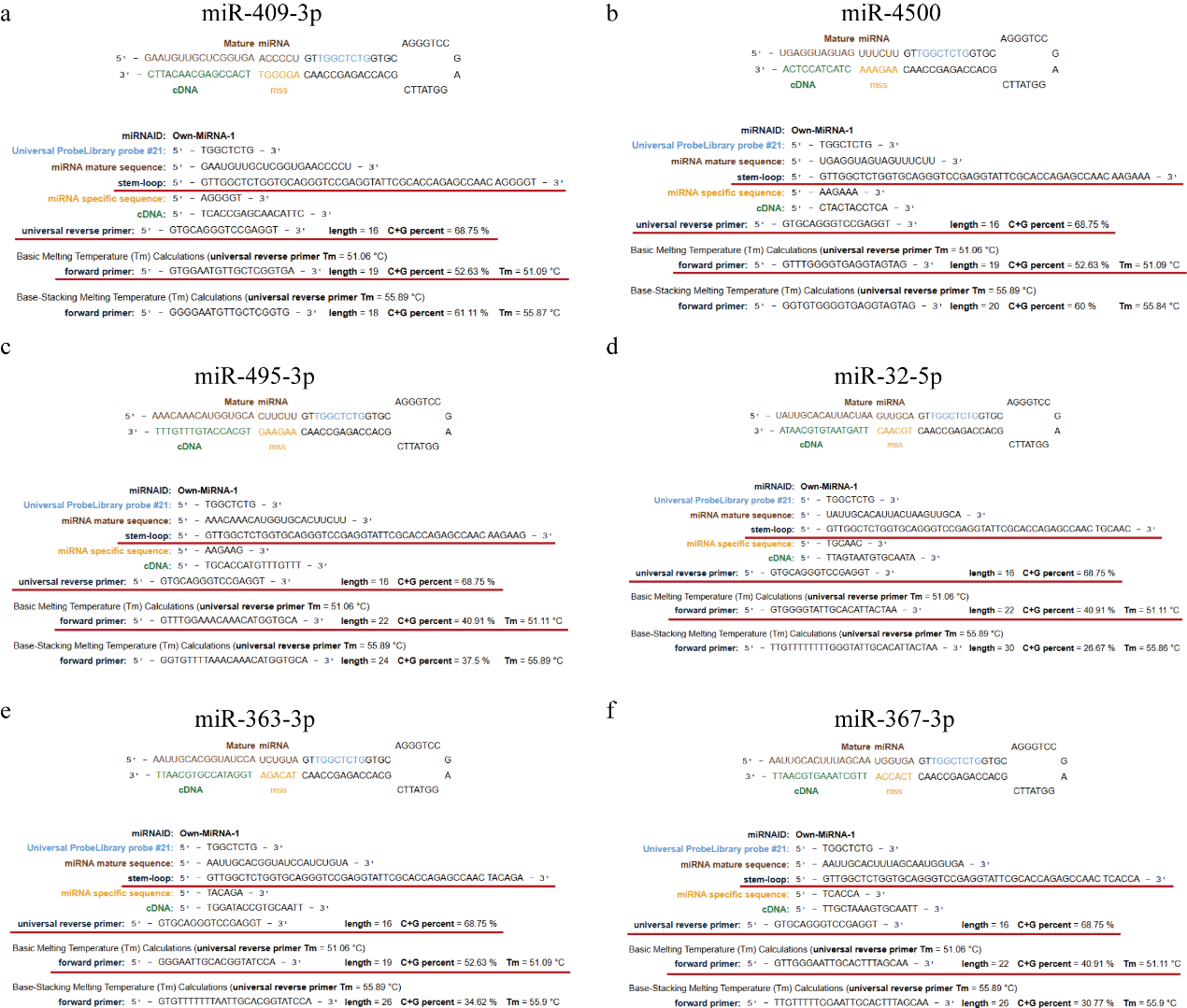
**Fig. S1** The stem-loop RT primer and the primers for qPCR assay were designed through miRNA Primer Design Tool.


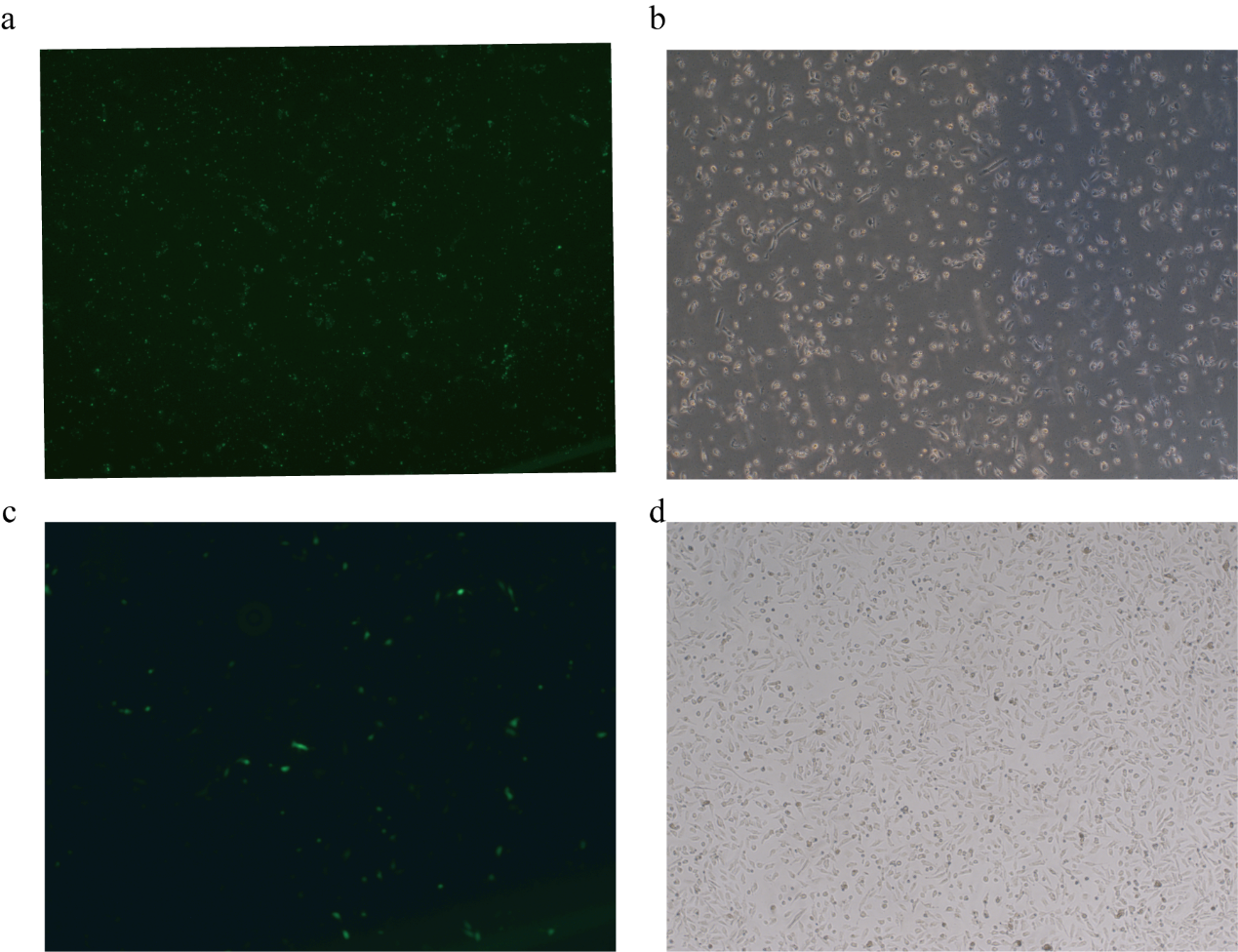


**Fig. S2** The transfection efficiency was detected through fluorescence microscope. **a, b**. The FAM was transfected into Ln229 cells and imaged under fluorescence microscope. **c, d.** The pcDNA3.1-EGFP plasmid was transfected into Ln229 cells and imaged under fluorescence microscope.


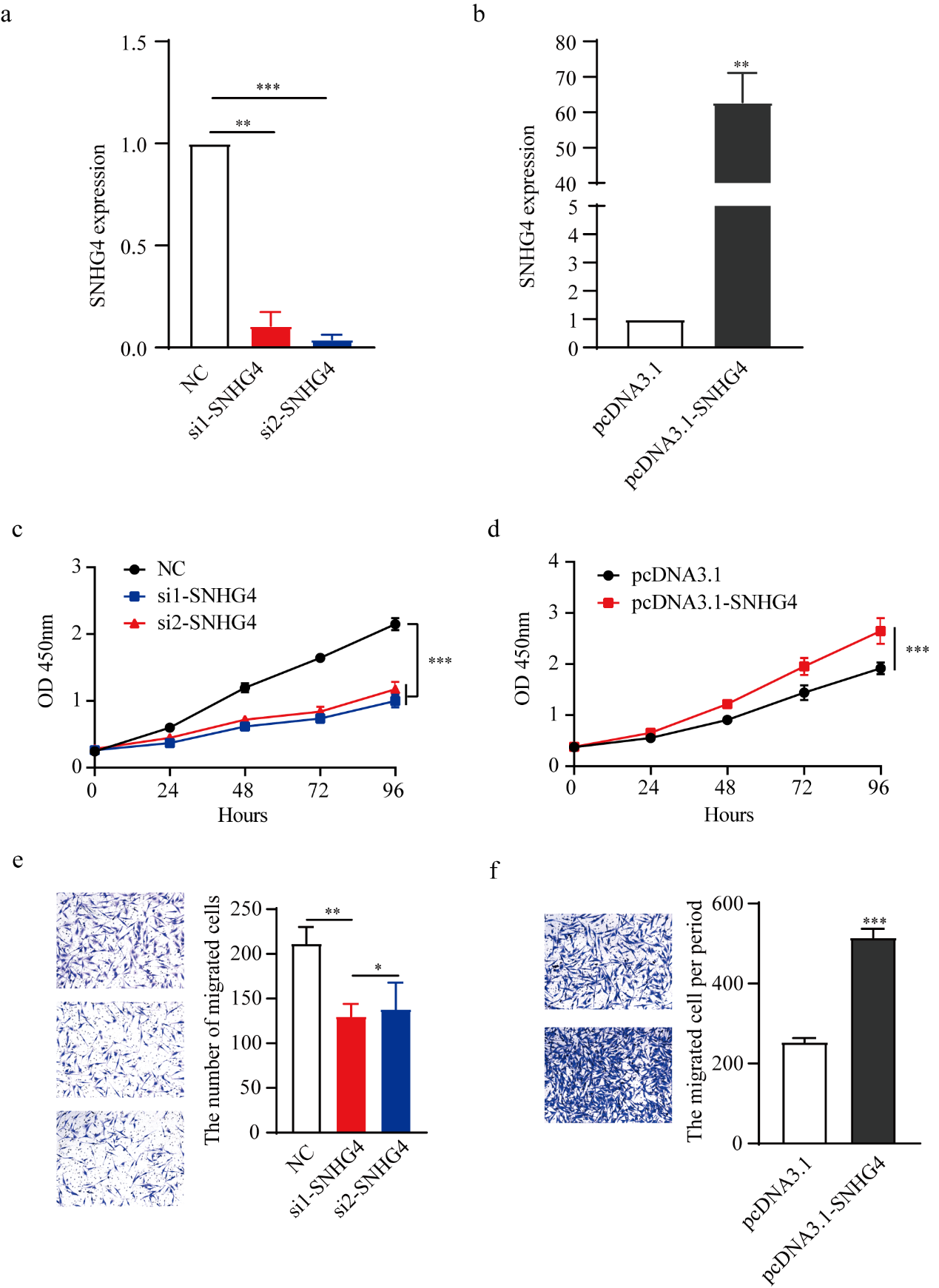


**Fig. S3** SNHG4 promoted the proliferation, migration of glioma cells *in vitro*. **a.** The knockdown efficiency of SNHG4 was detected in A172 cells by qRT-PCR assay when transfection with si1-SNHG4, si2-SNHG4 and NC. **b.** The overexpression efficiency of SNHG4 was detected in A172 cells by qRT-PCR assay when transfection with pcDNA3.1-SNHG4 plasmids. **c.** CCK-8 assay were used to assess the proliferation of A172 cells after knocking-down SNHG4. **d.** For overexpression of SNHG4 in A172 cells, the CCK-8 assay was used to assess the cell proliferation. **e.** Transwell assays were performed to examine cell migration in A172 cells transfected with SNHG4 siRNAs. **f.** Transwell assays were performed to examine cell migration in A172 cells when transfection with pcDNA3.1-SNHG4 plasmids. *: P <0.05, **: P <0.01, ***: P <0.001.


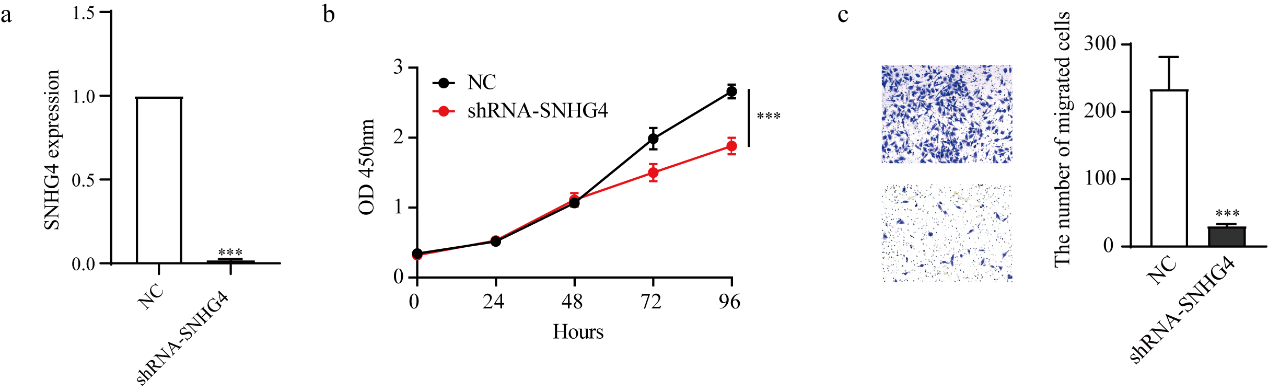


**Fig. S4** SNHG4 promoted the proliferation, migration of glioma cells *in vitro*. **a.** The knockdown efficiency of SNHG4 was detected in Ln229 cells by qRT-PCR assay when transfection with shRNA-SNHG4 and NC. **b.** CCK-8 assay were used to assess the proliferation of Ln229 cells after transfection with shRNA-SNHG4. **c.** Transwell assays were performed to examine cell migration in Ln229 cells transfected with shRNA-SNHG4. ***: P <0.001.


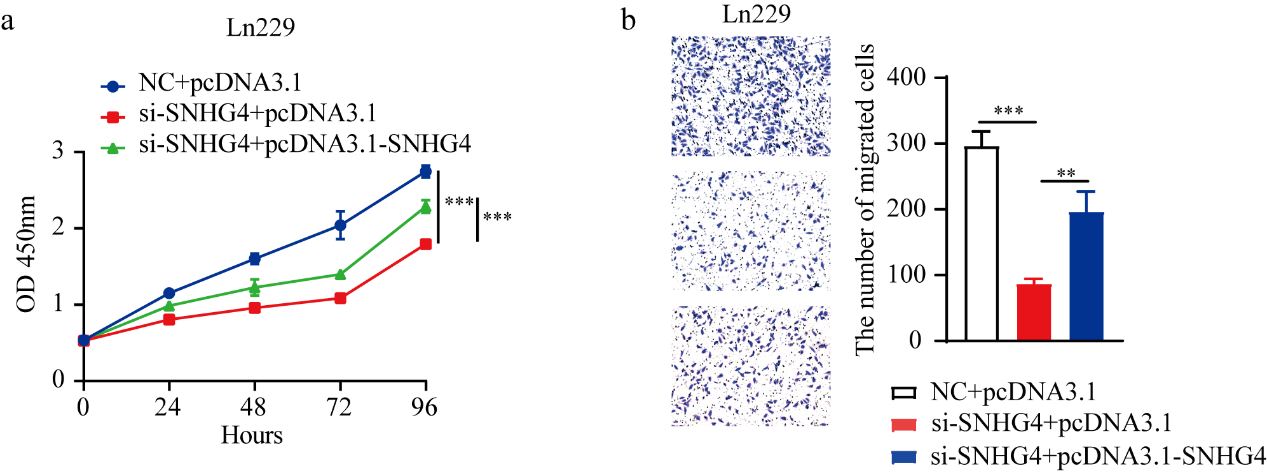


**Fig. S5** SNHG4 promoted the proliferation, migration of glioma cells through SNHG4 *in vitro*. **a.** CCK-8 assay were used to assess the proliferation of Ln229 cells after transfection with si-SNHG4, meanwhile transfected with pcDNA3.1-SNHG4. **b.** Transwell assays were performed to examine cell migration in Ln229 cells that transfected with si-SNHG4, meanwhile transfected with pcDNA3.1-SNHG4. **: P <0.01, ***: P <0.001.


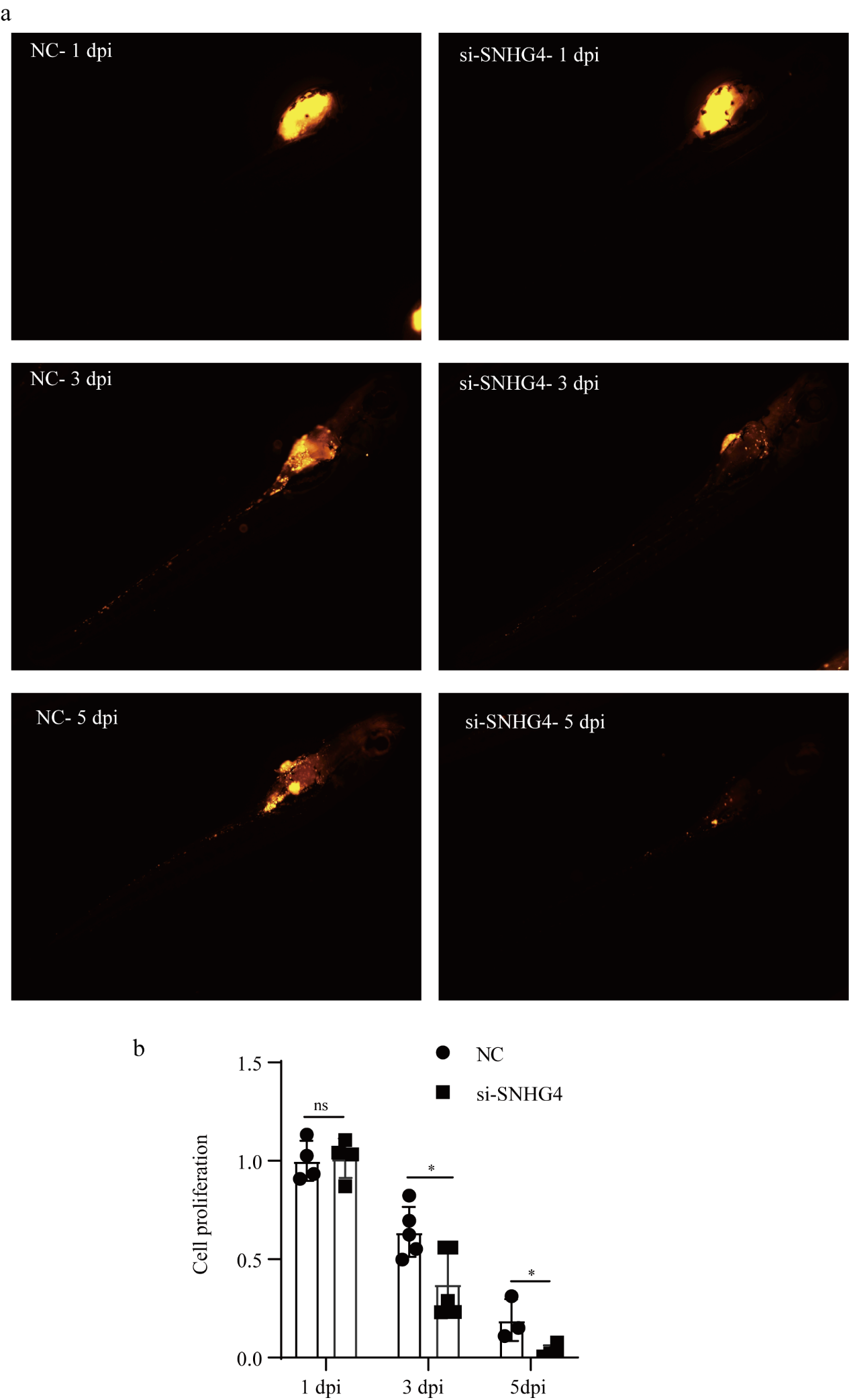


**Fig. S6** Knockdown of SNHG4 affects glioma progression in zebrafish xenograft. **a.** The glioma cells transfected with si-SNHG4 or NC were injected into the PVS of 2-dpf wild type zebrafish larvae. Images of the yolk were taken by a stereomicroscope at 1, 3, 5 dpi. **b.** The CM-DiI-positive areas in the yolk were quantified for cell proliferation. *: P <0.05


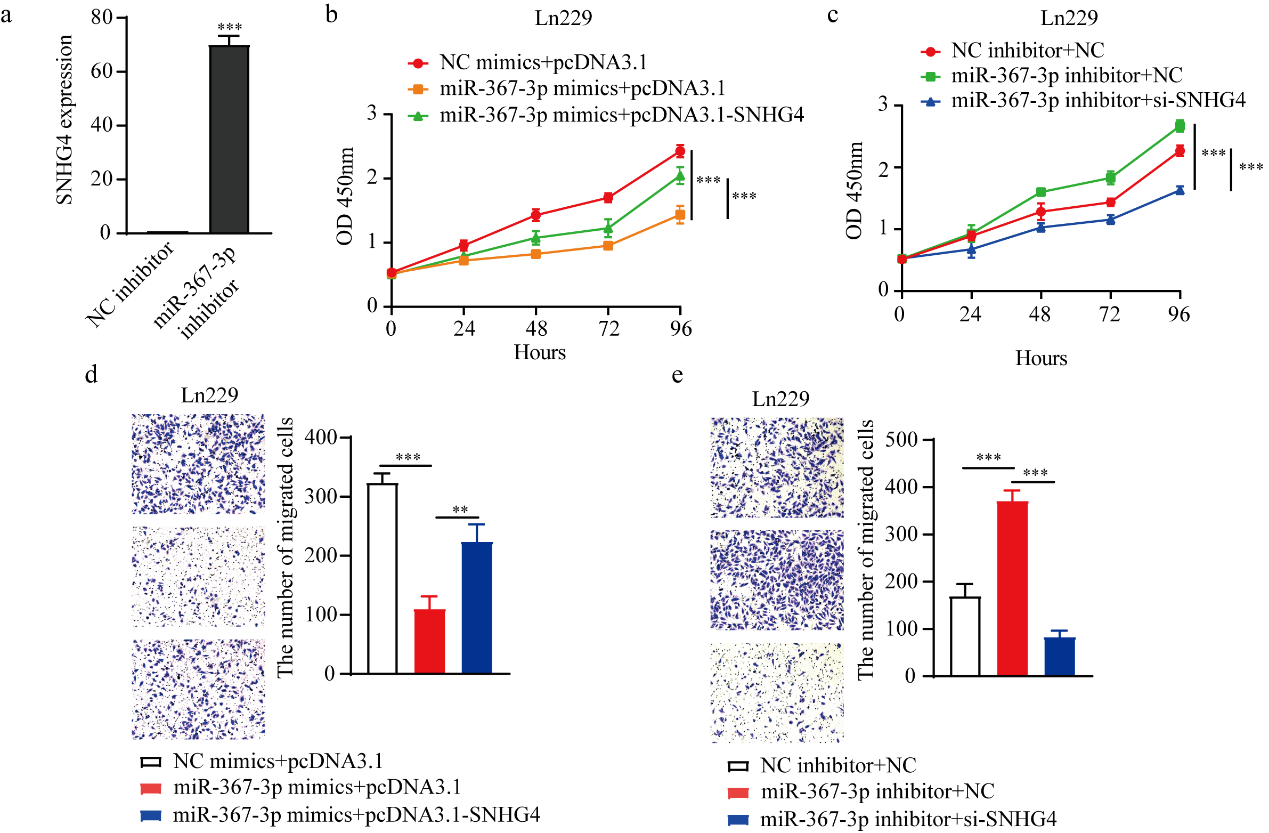


**Fig. S7** MiR-367-3p inhibited the proliferation, migration of glioma cells through SNHG4 *in vitro*. **a.** The SNHG4 expression was detected after transfection with miR-367-3p inhibitor in Ln229 cells. **b.** CCK-8 assay was used to assess the proliferation of Ln229 cells after transfection with miR-367-3p mimics, meanwhile transfected with pcDNA3.1-SNHG4. **c.** CCK-8 assay was used to assess the proliferation of Ln229 cells after transfection with miR-367-3p inhibitor, meanwhile transfected with si-SNHG4. **d.** Transwell assays were performed to examine cell migration in Ln229 cells that transfected with miR-367-3p mimics, meanwhile transfected with pcDNA3.1-SNHG4. **e.** Transwell assays were performed to examine cell migration in Ln229 cells that transfected with miR-367-3p inhibitor, meanwhile transfected with si-SNHG4. **: P <0.01, ***: P <0.001.


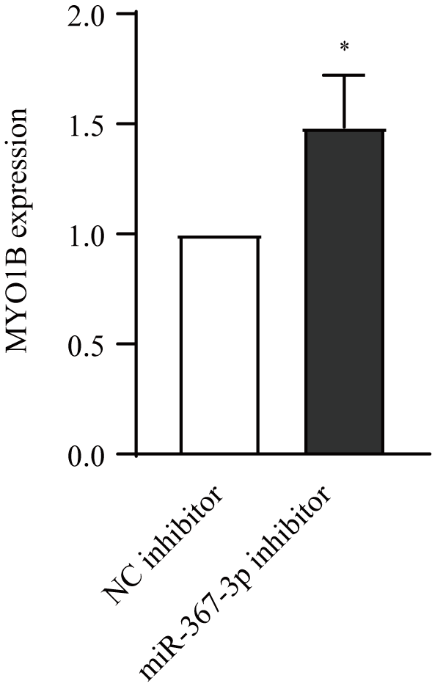


**Fig. S8** The expression level of MYO1B was detected after transfection with miR-367-3p inhibitor in Ln229 cells. *: P <0.05.


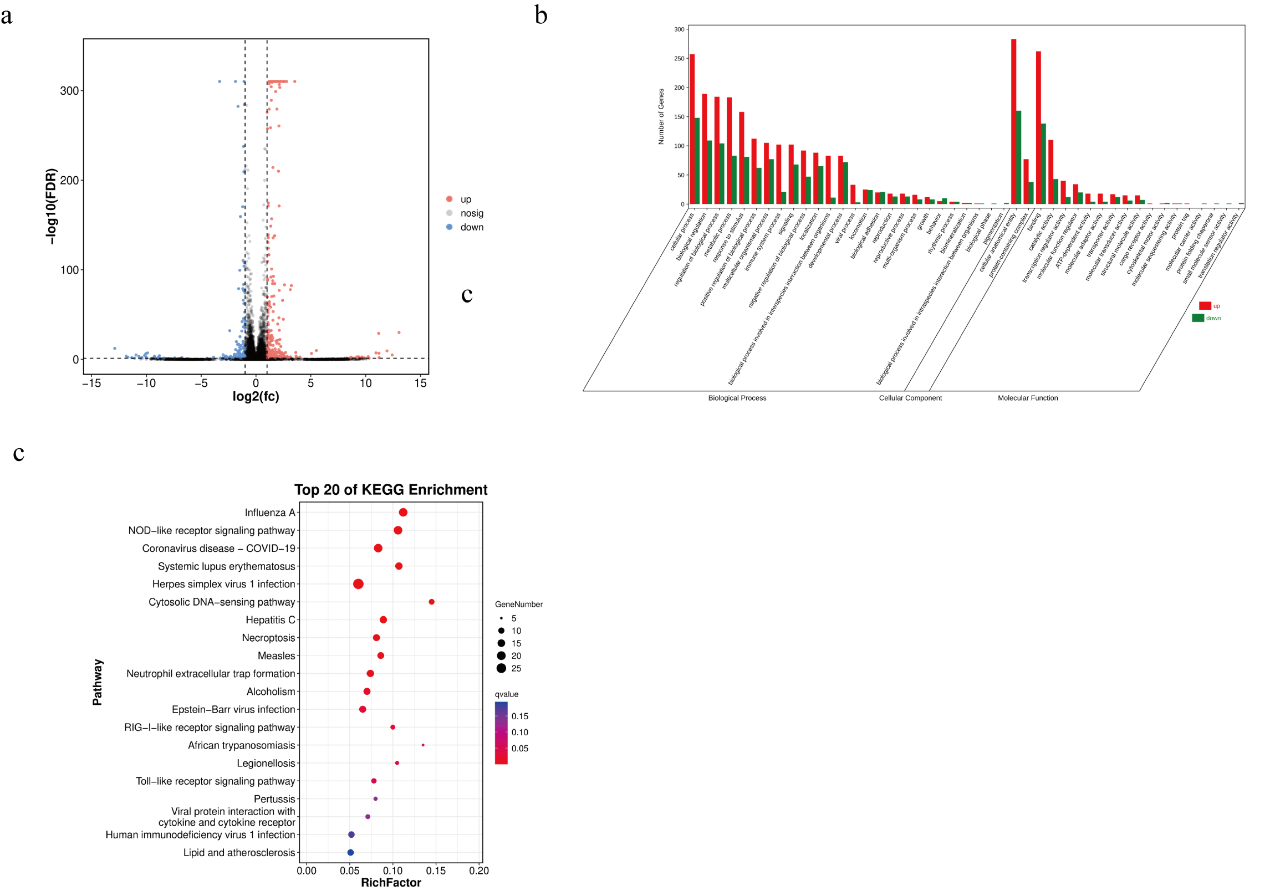


**Fig. S9** Analysis of RNA sequencing data after knockdown of SNHG4 in Ln229 cells. **a.** The volcano plot analysis based on the significant differences in genes among the comparison groups. **b.** GO enrichment classification bar chart. **c.** KEGG enrichment bubble chart.

The expression level of MYO1B was detected after transfection with miR-367-3p inhibitor in Ln229 cells. *: P <0.05.
